# *Gondysia* preceded *Neadysgonia* (Lepidoptera, Erebidae, Erebinae), a new generic synonymy from southeastern United States

**DOI:** 10.3897/zookeys.149.1747

**Published:** 2011-11-24

**Authors:** J. Bolling Sullivan, Albert Legrain

**Affiliations:** 1 200 Craven St., Beaufort, North Carolina 28516 USA; 2 Quai du Halage, 10, 4681-Hermalle (Liège), Belgium

**Keywords:** Taxonomy, *Dysgonia*, *Gondysia*, *Neadysgonia*, Madagascar, United States

## Abstract

The recently proposed genus *Neadysgonia* Sullivan, 2010, was preceded in the literature by *Gondysia* Berio, 1955, a monotypic genus based on specimens without locality labels but presumed to be from Madagascar. The genus *Gondysia* replaces *Neadysgonia* and the species *Gondysia pertorrida* Berio, 1955, becomes a junior synonym of *Gondysia consobrina* (Guenée, 1852).

## Introduction

Recently, the genus *Neadysgonia* Sullivan was proposed for the North American species formerly placed in *Dysgonia* Hübner (Sullivan 2010). Berio (1955) described the monotypic genus *Gondysia* based on material presumed to be from Madagascar. Superficially, *Gondysia pertorrida* Berio appears to be identical to specimens of *Neadysgonia consobrina* (Guenée). The purpose of this paper is to determine whether or not this possible synonymy is correct and, if so, to determine the taxonomic consequences of that finding.

## Materials and methods

During a visit to the British Museum in September of 2010 the type specimens of *Gondysia pertorrida*, their attached labels, and dissected genitalia, were photographed using a Cannon G10 camera and light box.

### Repository abbreviations

BMNH	Natural History Museum [statutorially: British Museum (Natural History)], London, UK
                

## Discussion

**Figure 1.**
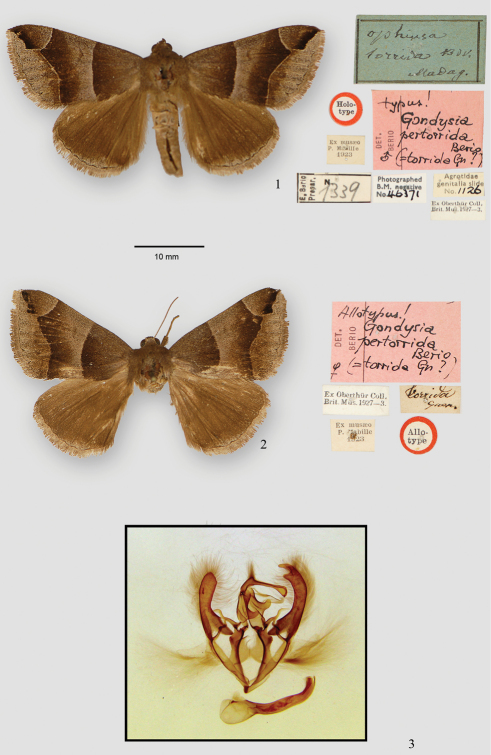
Male and female types of *Gondysia pertorrida* Berio and their affixed labels. Genitalia (BMNH slide: Agrotis 1126; E. Berio 1339) of male holotype of *Gondysia pertorrida* Berio.

During a visit to the BMNH we were able to examine the type specimens on which the name *Gondysia pertorrida* Berio is based. Adults were photographed as well as a slide preparation of the male genitalia of the type. The male and female of *Gondysia pertorrida*, with their labels, and the genitalia of the male holotype, are shown in Figure 1. British Museum records indicate that these specimens were obtained from M. P. Mabille who had a large amount of material from Madagascar and described many species from the island (bibliography in Poole 1989). The two type specimens in question were apparently obtained by Mabille via Oberthür. A. Guenée described most of the species currently in *Neadysgonia* but the types of all four of Guenée’s species are missing. There is no indication of a collecting locality on the labels of the two types of *Gondysia pertorrida*, but if these are the missing types for *Neadysgonia consobrina* as discussed in Sullivan (2010), the location was probably near Savanna, Georgia, USA.

Figures 6, 12 in Berio (1955) are line drawings of the uncus and tegumen of *Gondysia pertorrida* and are based on the genitalic preparation from the holotype in the BMNH. These closely resemble Figure 8a in Sullivan (2010) and examination of the slide in the BMNH (*Agrotis* 1126; E. Berio 1339) leaves no doubt that the type specimen of *Gondysia pertorrida* Berio is conspecific with our concept of *Neadysgonia consobrina* (Guenée). All of the key characters are present.

*Ophiusa consobrina* Guenée was described from an unknown number of specimens from an unknown locality. No type material was found in the BMNH collections or those of the Paris Museum (Sullivan 2010). *Neadysgonia consobrina* is a very uncommon species throughout most of its range and it is not unlikely that *Gondysia pertorrida* was described from the lost Guenée types.

*Gondysia pertorrida*, syn. n., is conspecific with *Neadysgonia consobrina* and thus is a junior synonym. However, the generic name *Gondysia* Berio is available and replaces *Neadysgonia* Sullivan, which is 55 years its’ junior. Therefore, the North American species are as follows:

*Gondysia* Berio, 1955

*Parallelia*, Auct. *nec* Hübner, 1818

*Neadysgonia* Sullivan, 2010, syn. n.

*Neadysgonia consobrina* (Guenée, 1852), syn. n.

*Neadysgonia redditura* (Walker, 1858)

*Neadysgonia pertorrida* Berio, 1955, syn. n.

*Neadysgonia similis* (Guenée, 1852), syn. n.

*Neadysgonia apicalis* (Guenée, 1852)

*Neadysgonia concolor* (Grote, 1893)

*Neadysgonia smithii* (Guenée, 1852), syn. n.

*Neadysgonia telma* (Sullivan, 2010), syn. n.

Recent collecting and genitalic examination have extended the known range of *Gondysia smithii* to northern Florida, *Gondysia telma* to central Florida (Terhune Dickel pers. comm.), and of *Gondysia smithii* to Virginia (Steve Roble, pers. comm.).
